# Contributions of Health Psychology to Climate Change: A Review

**DOI:** 10.3390/ijerph22040634

**Published:** 2025-04-17

**Authors:** Elisa Kern de Castro, Marta Reis

**Affiliations:** 1 Egas Moniz Center for Interdisciplinary Research, Egas Moniz School of Health and Science, 2829-511 Almada, Portugal; 2 Egas Moniz School of Health and Science, 2829-511 Almada, Portugal; mreis@egasmoniz.edu.pt; 3 Faculdade de Motricidade Humana, Universidade de Lisboa, 1495-751 Lisbon, Portugal; 4 ISAMB/Faculdade de Medicina, Universidade de Lisboa, 1649-026 Lisboa, Portugal

**Keywords:** health psychology, climate change, behavior change, mental health, sustainability

## Abstract

Climate change poses a significant threat to human health, necessitating interdisciplinary approaches to mitigate its effects. Health psychology, with its focus on behavior change and well-being, is uniquely positioned to contribute to climate action. This review examines how health psychology can address climate-related challenges, emphasizing psychological responses to environmental stressors, behavior modification strategies, and public health interventions. The findings indicate that climate change functions as a psychological stressor, contributing to anxiety, depression, and eco-distress. Additionally, behavioral science insights are underutilized in climate governance, despite their potential to drive sustainable actions. Health psychology can enhance climate adaptation by promoting pro-environmental behaviors, fostering resilience, and integrating psychological well-being into policy frameworks. However, barriers such as limited interdisciplinary collaboration and insufficient emphasis on systemic change hinder progress. To maximize impact, health psychologists must engage in climate governance, advocate for policy integration, and address both individual and collective behavior change. Future research should explore the intersection of mental health, climate resilience, and behavior adaptation to develop comprehensive strategies for tackling climate change. This review underscores the urgent need for health psychology to play a more active role in shaping climate policy and fostering sustainable, health-promoting behaviors.

## 1. Introduction

The historical understanding of health, illness, and human beings has been limited and fragmented, traditionally conceptualizing the mind and body as distinct entities. This perspective has been largely shaped by the biomedical model, which explains illness independently of its social context [1,2]. The transition from the biomedical paradigm to the biopsychosocial paradigm of health is gradual and complex, necessitating the incorporation of psychological and social dimensions into health science. The emergence of this paradigm has fostered a more holistic and integrative approach to health, emphasizing well-being and quality of life [1,2].

The One Health approach assumes that human health is closely related to the health of our surrounding environments, including animals, plants, and the ecosystems on which we live and depend [3]. Ecosystem degradation and climate change are predominantly anthropogenic in origin; thus, mitigating these threats requires profound and sustained behavioral change [4]. However, behavioral science has been largely overlooked in research on biodiversity conservation, environmental degradation, and climate change. Furthermore, existing psychological insights on these topics remain underutilized [4]. Addressing these global challenges necessitates interdisciplinary collaboration, integrating diverse fields of knowledge to advance solutions for planetary and human well-being.

Over the past two decades, psychology has increasingly intersected with ecology and climate change. Various subfields of psychology contribute unique perspectives, including ecopsychology [5], research on the mental health impacts of climate change [6] and natural disasters, and studies on behavioral adaptation to environmental shifts [7]. Additionally, environmental psychology has made significant contributions by exploring the effects of the environment on human well-being, the relationship between humans and nature, and the factors that influence pro-environmental behavior. This interdisciplinary convergence underscores the critical role of psychological science in understanding and promoting ecological sustainability [5]. The integration of these perspectives is essential for developing a comprehensive understanding of psychological reactions and behaviors toward climate change and for designing effective interventions to foster more sustainable behaviors.

In this context, psychology must avoid framing its expertise on crisis and stress solely within an individualistic or medicalized paradigm [8]. The paradigm shift from the biomedical to the biopsychosocial model [9] has underscored the vital contributions of health psychology. These contributions are particularly evident in research on health behaviors, promoting healthy lifestyles, and disease prevention [9].

Given these considerations, the purpose of this paper is to examine how health psychology can address climate-related challenges, emphasizing psychological responses to environmental stressors, behavior modification strategies, and public health interventions. It posits that climate change constitutes a significant psychological stressor and, in this context, explores strategies for coping, fostering pro-environmental behavior, and promoting both health and resilience in response to this global threat.

## 2. Climate Crisis and Psychology

Climate change and planetary degradation are primarily anthropogenic challenges rather than merely environmental phenomena [10]. Society must undertake both immediate and long-term adaptive strategies to mitigate the effects of rising global temperatures. However, current efforts remain insufficient [11]. Research and investment in climate science have predominantly focused on the natural and physical sciences, often overlooking the human behaviors that drive environmental degradation on a large scale [10]. The consequences of climate change, including floods, droughts, and hurricanes, have already become evident [12,13]. Given these escalating threats, it is imperative to examine the factors that prevent individuals from altering their behaviors in response to the climate crisis or mobilizing to address its implications in their daily lives [10,14,15].

Psychology, as a discipline, seeks to understand human behavior, predicting and explaining how individuals adapt to their environments. It has traditionally approached this from an individual rather than a collective perspective, often assuming that human behavior is rational or at least intentional [8,16]. Climate change already influences human behavior and is expected to do so increasingly in the future. Conversely, health-related behaviors also significantly impact climate change, either through commitment to mitigation efforts or by contributing to pollution and carbon emissions [17]. However, many behaviors occur automatically, shaped by habits and routines that respond to environmental cues, often outside of conscious awareness [16]. As such, behavioral patterns are not always driven by deliberate decision-making [16].

Psychological science offers a broad repertoire of theoretical and intervention-based tools, spanning domains such as mental health, human behavior, interpersonal relationships, and decision-making processes, which could play a significant role in addressing climate-related challenges [18]. However, the same authors highlight that advancing climate action requires psychologists to critically evaluate their conceptual frameworks and adopt an interdisciplinary approach that contributes to effective climate governance.

The assumption that psychology’s primary role is to help individuals adapt to environmental changes may be inadequate in addressing the urgency of climate change. While resilience promotion and well-being protection are essential, they may also inadvertently mitigate the psychological burden of climate change without fostering substantive environmental action [13]. This could result in a focus on encouraging low-impact behaviors, such as energy conservation, rather than advocating for transformative systemic shifts, such as transitioning to renewable energy sources [16]. Addressing climate change requires profound behavioral changes at both the individual and societal levels [8]. Effective public policies could include strategies such as reducing car usage by providing free public transportation or implementing free school meal programs to decrease commuting-related emissions [11].

Adams (2021) [8] critiques the approach that frames climate change mitigation primarily in terms of modifying individual behaviors. He argues that such perspectives are reductionist, as they fail to account for broader social dynamics, power structures, discourse, and emotional factors. Predicting climate-related behaviors solely through individual behavior change models has led to ineffective interventions that prioritize informational campaigns over structural changes in mobility, food consumption, and pollution reduction [16]. Moreover, the impacts of climate change are unequally distributed, being influenced by local, economic, and cultural disparities, which perpetuate systemic injustices, particularly among marginalized communities [19]. Addressing these complexities necessitates refining psychological theories and intervention strategies to integrate individual-level, social, and structural dimensions relevant to climate change adaptation and mitigation [8].

A recent systematic review of the role of psychology in climate change governance [20] underscores that significant progress remains to be made in this area. While psychological research has provided valuable insights, particularly regarding personal adaptation and decision-making, the psychological distress induced by climate change—recognized as a fundamental aspect of the human experience—has been largely neglected in climate governance discussions. Furthermore, despite psychology’s incorporation into select studies, its role remains superficial within interdisciplinary research dominated by contributions from political science, environmental science, management, law, economics, and ecology. This highlights psychology’s untapped potential to contribute meaningfully to climate change discourse, presenting a critical and promising direction for future research and collaboration.

This article is organized into two primary sections, the second of which is further subdivided into subsections. Initially, the article undertakes a broad overview of the role of psychology in the climate crisis, with a particular emphasis on human behavior. The subsequent section delineates the specific role of health psychology in the context of the climate crisis, accentuating the climate crisis’s perception as a stress-inducing event and its subsequent repercussions on health. This section then delves into the topic of the climate crisis, stress, and mental health; the role of behavior and risk perception in the climate crisis; and, within this topic, the climate crisis and how to promote behavioral change and pro-environmental behavior. Concluding the analysis, the paper discusses the future implications of the role of health psychology in the climate crisis and climate change, and its contribution to the interdisciplinary field.

## 3. Climate Crisis and Health Psychology

Health psychology is a discipline that aims to contribute to the promotion and maintenance of health, the prevention and treatment of illnesses, and the development of health policies and improvements in healthcare systems [9]. It provides a comprehensive understanding of health and illness processes by adopting a biopsychosocial paradigm, which integrates biological, psychological, and social determinants of health. A central focus of health psychology is identifying the factors that lead individuals to engage in unhealthy behaviors and developing effective strategies to support long-term health maintenance [21].

Health psychology plays a crucial role in public health by facilitating disease prevention and risk reduction through both individual and collective behavioral changes [22]. Over time, the field has increasingly acknowledged the significance of social and environmental determinants of health, aligning with an ecological perspective [21]. Consequently, health outcomes are influenced not only by individual choices but also by broader environmental and societal conditions. One of the primary objectives of health psychology is to predict and modify unhealthy behaviors [23]. This focus establishes a direct connection between health psychology, pro-environmental behavior, environmental conservation, and climate change [11]. Climate change disproportionately affects vulnerable populations worldwide, exacerbating social inequalities and poverty, which must be considered when addressing its consequences and designing intervention strategies [8].

Despite its potential contributions, health psychology’s engagement with climate change remains limited [17]. Several factors contribute to this underrepresentation, including a lack of specialized education and training for health psychologists in climate-related issues, insufficient awareness of the urgency of climate change, hesitancy in exploring interdisciplinary research areas, and challenges in securing funding for such studies. Addressing these barriers is essential to enhancing health psychology’s role in climate change mitigation and adaptation efforts. Given the field’s expertise in behavioral change and health promotion, it holds significant potential to contribute meaningfully to climate action, emphasizing the need for increased research, training, and policy integration in this domain.

### 3.1. Climate Crisis as a Stressor Event and Its Repercussions on Health

The climate crisis is a significant stressor associated with adverse emotional and psychological responses. Initially, concerns regarding its effects were primarily centered on the potential diseases caused by pollution and environmental degradation [5]. However, over time, the impact of climate change on mental health and overall well-being has become increasingly evident. In health psychology, particularly within a clinical framework, the stress–vulnerability model provides a key theoretical perspective. This model posits that stress-related mental health disorders arise from the interaction between external stressors and an individual’s inherent vulnerability to stress [5,24]. From a psychological standpoint, stress occurs when internal or external environmental demands are perceived as threatening, unpleasant, or aversive. Such demands elicit a psychophysiological response that varies among individuals, influencing both psychological and physical health [24,25]. Stress responses are dynamic and flexible, shaped by an individual’s appraisal of the stressor, which can differ widely among people. Stressors, in turn, are defined as circumstances or events that endanger significant personal goals, such as physical integrity or psychological well-being, necessitating adaptation to new conditions [25].

The climate crisis represents a multifaceted stressor that elicits both acute and chronic stress responses in individuals and communities [18]. Certain climate-related stressors, such as natural disasters, trigger immediate and overwhelming psychological reactions, including trauma and shock. In contrast, slower-moving environmental changes, such as the gradual increase in global temperatures, contribute to persistent, low-grade stress that accumulates over time.

A range of environmental stressors associated with climate change have been identified, including both extreme climatic events (e.g., hurricanes and floods) and less acute but continuous stressors (e.g., rising global temperatures). These environmental changes have far-reaching consequences, affecting employment patterns, food security, transportation infrastructure, cultural identity, self-esteem, and an individual’s connection to their community and environment [26]. Additionally, the degradation of ecosystems and forced population displacement exacerbate these stressors, creating widespread societal disruptions.

The psychological impact of climate change extends beyond direct stressors, often resulting in profound emotional responses, including despair, sadness, and grief. These experiences can disrupt social relationships, weaken support networks, and heighten vulnerability among at-risk populations, such as individuals with pre-existing medical conditions, Indigenous communities, children, and the elderly [5,18]. Emerging research highlights the prevalence of climate anxiety, a condition characterized by fear, helplessness, and guilt related to climate change, particularly among younger generations [14,27]. This emotional burden not only affects mental health but also impairs individuals’ ability to engage in adaptive coping behaviors.

From an existential perspective, the climate crisis introduces novel psychological constructs such as ecological grief, which describes the sorrow and distress associated with environmental losses, whether anticipated or realized [28]. The crisis forces individuals to confront a heightened awareness of mortality, not only on a personal level but also as a species-wide existential threat, resulting in significant psychological distress [5,18,20,28].

Thus, climate change has both direct and indirect effects on health, influencing both physical and mental well-being. Addressing its consequences requires an interdisciplinary approach that integrates psychological, public health, and environmental science perspectives to develop effective mitigation and adaptation strategies [17,28].

### 3.2. Climate Crisis, Stress, and Mental Health

The climate crisis is a pervasive and multifaceted stressor that significantly impacts mental health and psychological well-being. Within the framework of health psychology, stress is conceptualized as a psychophysiological response to external or internal demands perceived as threatening or overwhelming [24]. Climate change introduces both acute stressors, such as extreme weather events, and chronic stressors, including long-term environmental degradation and socio-economic instability, which collectively influence individuals and communities [12,17,26].

Acute stressors, such as hurricanes, wildfires, and flooding, frequently lead to immediate trauma responses, including post-traumatic stress disorder (PTSD), among survivors. These events disrupt daily life, displace populations, and weaken social support networks, contributing to widespread psychological distress [26]. In contrast, chronic stressors, such as rising sea levels, prolonged droughts, and heat waves, exert a more insidious effect on mental health. These persistent environmental changes are associated with heightened rates of anxiety, depression, and feelings of helplessness, particularly in communities experiencing cumulative socio-environmental adversity [5].

Despite well-documented links between climate change, stress, and mental health, these relationships remain underrepresented in scientific discourse. A critical distinction must be made between individuals who experience direct exposure to climate-related events and those who suffer from indirect psychological consequences [10,14]. People directly affected by extreme climate events—such as wildfires, hurricanes, or floods—often experience acute stress reactions and trauma, whereas individuals indirectly affected may still develop climate anxiety and existential distress stemming from their awareness of the crisis and its long-term implications [10,29].

When an individual’s response to a climate-related stressor is accompanied by a sense of helplessness or intense fear—triggered by a perceived threat to life or well-being—the psychological reaction may resemble trauma [30]. Extensive research on trauma has examined the adverse psychological effects of extreme stress, particularly PTSD, which manifests through intrusive memories, avoidance behaviors, negative alterations in cognition and mood, and heightened physiological reactivity [31,32].

Climate events can be categorized into three categories: acute, sub-acute, and persistent [6]. Acute climate events (e.g., wildfires, hurricanes, and floods) trigger immediate psychological reactions, often resulting in acute stress disorder, PTSD, anxiety, depression, and sleep disturbances. These effects can persist for months or even years, depending on the severity of exposure and individual vulnerability. Identified risk factors for the development of these disorders include the magnitude of the climate event, loss of a loved one, female gender, low socioeconomic status, lower education level, ethnic minority status, young age, limited social support, and a family history of mental health conditions [6]. Sub-acute climate events, including heatwaves and droughts, have been linked to increased aggression, delinquent behavior, and elevated suicide rates, particularly among men and older individuals. Prolonged exposure to extreme heat has also been associated with hormonal imbalances affecting thyroid function, which can manifest as mood disturbances, cognitive impairments, and lethargy. Droughts have been correlated with a heightened prevalence of depression and suicide in rural populations. Finally, persistent climate-related phenomena (e.g., rising sea levels and increasing global temperatures) contribute to widespread societal distress. The uncertainty surrounding the future and concerns about planetary habitability generate chronic anxiety, existential dread, and ecological grief [6]. Table 1 provides information about the key psychological constructs related to the climate crisis.

Beyond the stress–trauma paradigm, climate change is increasingly recognized as an existential threat that disrupts psychological well-being and human development [5,18,20]. On a broader scale, the mental health repercussions of climate change can be interpreted through the lens of a dysfunctional relationship between humans and the natural environment. This imbalance fuels ecological crises, which in turn drive psychological distress [5].

The ongoing climate crisis fosters insecurity and uncertainty regarding the future, contributing to climate anxiety psychological response characterized by fear, distress, and perceived helplessness in the face of environmental degradation [14]. While anxiety in uncertain situations is not inherently pathological, it can become clinically significant when it interferes with daily functioning, disrupting sleep, work performance, and social engagement. Severe climate anxiety may also contribute to identity disturbances and a diminished sense of belonging, particularly among individuals displaced by climate-related disasters, leading to profound grief and loss [14].

Notably, climate anxiety is not restricted to those who have directly experienced environmental disasters. It can arise from subjective perceptions of climate threats, heightened awareness of ecological risks, and perceived personal vulnerability. A recent systematic review examining the mental health of young adults in relation to climate change [33] highlighted that most research focuses on individuals diagnosed with anxiety or PTSD following direct exposure to extreme climate events. However, studies exploring the psychological effects of climate change on the general population remain scarce, particularly regarding individuals who experience persistent, indirect stress due to climate concerns. The review identified protective factors against climate-related distress, including strong family support, robust social networks, and cultural identity. Conversely, avoidance-based coping strategies were found to be ineffective in promoting psychological adaptation. Moreover, research on climate change’s mental health effects remains disproportionately focused on industrialized Western nations, with limited studies addressing the experiences of vulnerable minority groups, such as Indigenous populations and migrant communities, who face heightened exposure to climate-related stressors.

Efforts to mitigate climate anxiety and promote psychological well-being are crucial in addressing the mental health dimensions of climate change. Clayton [14] suggests that individuals can benefit from strategies aimed at managing emotional responses, cognitive appraisals, and behavioral adaptations to climate threats. However, this presents a paradox: problem-focused coping strategies, which aim to resolve stressors, may increase psychological distress when applied to climate change, given the vast and systemic nature of the crisis. Conversely, cognitive reframing techniques that alter subjective perceptions of climate threats may reduce anxiety but risk minimizing the perceived urgency of the crisis, potentially undermining motivation for climate action.

Emotions play a central role in climate adaptation and mitigation efforts. Psychological responses such as fear, anticipatory grief, ecological grief, dread, despair, existential crises, and trauma linked to extreme weather events shape individual and collective reactions to environmental challenges [8]. From a trauma-informed perspective, catastrophic climate events—such as the severe flooding in southern Brazil [34] and Valencia, Spain [35]—can be understood as collective psychological stressors requiring systemic intervention. Furthermore, the geographic and social distribution of climate-related distress reveals underlying inequalities, demonstrating that marginalized populations disproportionately bear the psychological burden of climate change [8].

Addressing the mental health impacts of climate change necessitates a holistic and interdisciplinary approach. Psychological interventions, including trauma-focused therapy and community-based resilience programs, can facilitate adaptive coping and enhance psychological resilience in individuals and groups facing acute and chronic climate stressors [36]. Additionally, integrating mental health considerations into climate adaptation and mitigation policies is essential to fostering societal resilience. By prioritizing mental health alongside environmental and physical well-being, societies can enhance their capacity to navigate the evolving challenges posed by climate change. Strengthening social support networks, promoting pro-environmental behavior, and fostering adaptive coping mechanisms are critical steps in mitigating the psychological toll of an increasingly unstable climate. Table 2 summarizes the climate-related events and associated psychological outcomes.

### 3.3. Climate Crisis, Behavior, and Risk Perception

Climate change constitutes a significant public health emergency. Variations in weather patterns, including droughts and floods, disrupt agricultural production, alter nutrient cycles, and have both direct and indirect effects on human well-being. Soil degradation further impacts the nutritional composition of food, contributing to physical health challenges while exacerbating mental health conditions such as anxiety, depression, and stress [12].

The risks associated with climate change have a cascading effect, influencing psychological, social, and behavioral dynamics. A substantial body of psychological research has identified key social, cognitive, and motivational factors that shape public attitudes toward climate change, including climate skepticism [15]. Among the most salient determinants of climate attitudes are personal values, cultural worldviews, political affiliations, and ideological beliefs [27]. Political polarization has been widely studied as a significant barrier to the implementation of effective climate policies and behavioral change [15]. However, research on the psychosocial consequences of climate change remains limited, with most studies focusing on all-cause mortality and climate-related physical health effects, such as respiratory and cardiovascular diseases [3].

A critical determinant of an individual’s willingness to engage in climate action is their belief in climate change [27,35]. Climate belief systems encompass three interrelated but distinct dimensions: [1] acknowledgment of climate change as a real phenomenon, [2] attribution of climate change to anthropogenic activities, and [3] perception of climate change as primarily detrimental. Individuals who accept the reality of climate change are more likely to attribute it to human activities and recognize its predominantly adverse consequences. These beliefs indirectly influence climate-related behaviors by increasing individuals’ perceptions of specific climate risks (e.g., flooding), reinforcing their sense of personal responsibility, and strengthening moral obligations to act [27].

Climate change denial can be driven by multiple psychological mechanisms, including cognitive dissonance, mortality awareness, and system justification [21]. The recognition that climate mitigation requires collective action can lead to a sense of powerlessness, discouraging individual engagement. Consequently, some individuals may resolve this cognitive dissonance by rejecting the severity of climate change or denying personal responsibility for mitigation efforts. Moreover, political and governmental inaction can reinforce public perceptions that climate change is not a pressing concern, reducing individuals’ motivation to engage in pro-environmental behaviors [21].

Despite widespread acknowledgment of climate change, a critical challenge lies in understanding why many individuals fail to act on their beliefs [27]. One explanation is the perception that adaptation, without mitigation, is sufficient to address climate-related threats. Another contributing factor is eco-paralysis, a state of inaction arising from the perception that neither individual nor governmental efforts are sufficient to address the magnitude of the climate crisis [21]. Additionally, the unequal distribution of climate change consequences exacerbates feelings of injustice, guilt, and anger, particularly among populations disproportionately affected by environmental degradation despite contributing minimally to global emissions.

Conversely, individuals may engage in pro-environmental behaviors when these actions align with their personal values. People motivated by altruistic values prioritize societal well-being and equity, while those with strong biospheric values prioritize environmental conservation [27]. However, individuals may also adopt pro-environmental behaviors for self-serving reasons, such as financial benefits or social status enhancement.

While most individuals care about climate change, they often underestimate the extent to which others share their concerns and engage in climate action. This misperception can serve as a barrier to collective action. Additionally, external constraints, such as infrastructural limitations and lack of governmental support, can impede consistent engagement in climate-positive behaviors. Addressing these barriers requires systemic changes that facilitate large-scale climate action [27].

In health psychology, risk perception refers to an individual’s subjective evaluation of their susceptibility to threats, informed by cognitive assessments, emotional responses, social influences, and cultural norms [37,38]. While awareness of objective risks does not necessarily drive protective behaviors, specific cognitive and emotional processes mediate behavioral responses [39]. Notably, individuals tend to underestimate risks when comparing their vulnerability to that of others, leading to discrepancies between perceived and actual threats [26,38].

Within the context of climate adaptation, risk perception and perceived adaptive capacity play pivotal roles in shaping individual and household-level adaptation behaviors [26]. However, additional factors—such as place attachment, trust in government, climate knowledge, and perceived responsibility—significantly influence adaptation efforts [27]. A recent meta-analysis [15] found that actual adaptation behaviors were most strongly associated with negative affect, social norms, perceived self-efficacy, and perceived response efficacy. In contrast, prior experience with climate-related events and knowledge about adaptation strategies exhibited only weak correlations with actual adaptation behaviors.

Climate change is a multifaceted phenomenon that many individuals struggle to comprehend fully, given the intricate interactions between human activities and global climate systems [7]. Humans tend to prioritize immediate concerns over long-term risks, a bias compounded by the salience of extreme weather events, which are more tangible than gradual climatic shifts. Consequently, individuals may react strongly to specific disasters, such as hurricanes or wildfires, while failing to perceive climate change as an overarching existential threat.

Psychological and ideological factors further contribute to climate denial. System justification theory suggests that individuals are motivated to defend existing social and economic systems, making it difficult to accept that Western lifestyles significantly contribute to climate change. Similarly, within some religious communities, belief in a benevolent and omnipotent deity may conflict with the scientific consensus on climate change, leading to resistance against climate action. Additionally, financial and political interests often contribute to public misinformation, fostering confusion and inaction. Pluralistic ignorance—wherein individuals collectively underestimate the extent to which others recognize and prioritize climate change—further exacerbates climate inaction. In some cases, climate denial extends beyond personal belief systems and becomes embedded in social identity [37].

The influence of values on behavior is mediated by cognitive and emotional processes. Emotions act as signals that indicate when values are threatened (eliciting negative emotions such as fear or guilt) or upheld (eliciting positive emotions such as pride or fulfillment). However, risk perception remains a critical moderating factor. Individuals who strongly identify with biospheric values but experience heightened climate-related fear may disengage from the issue rather than take action [37].

The probability that individuals will engage in climate mitigation behaviors and offer their endorsement to mitigation policies is contingent upon their belief, the human-caused nature of climate change, and the deleterious consequences of climate change. Furthermore, people are more likely to adopt adaptive behaviors and endorse climate adaptation policies when they perceive climate change as a tangible, human-driven, and menacing phenomenon [27]. This underscores the complex interplay between psychological processes, risk perception, and behavioral responses to climate change. Addressing these challenges requires interdisciplinary collaboration between psychologists, policymakers, and environmental scientists to design interventions that foster adaptive behaviors and enhance public engagement with climate action [7]. As shown in Table 3, promoting climate engagement requires addressing emotional, ideological, and cognitive barriers at both individual and societal levels.

### 3.4. Climate Crisis and How to Promote Behavior Change and Pro-Environmental Behavior

Climate change represents a critical global challenge that necessitates behavioral shifts at individual and societal levels. Psychology plays a pivotal role in facilitating the transition from risk awareness to adaptive action, enabling individuals and communities to move beyond knowledge toward meaningful behavioral change [7,11,12,15,17].

In health psychology, healthy behavior is central to promoting well-being, preventing disease, detecting illness in its early stages, maintaining health, and aiding recovery [20]. From an ecological perspective, however, not all healthy behaviors are sustainable. While engaging in sports is undoubtedly beneficial to one’s health, the practice of motorized sports, for instance, is not sustainable due to their substantial carbon emissions [14]. To address this disconnect, researchers advocate for an expanded conceptualization of healthy behavior that incorporates environmental determinants and consequences. In this sense, the present approach may be regarded as a component of the planetary health approach. The latter is an approach that seeks to link human health and planetary ecosystems, based on the idea that disruption of the Earth’s systems, including climate change, has direct and indirect effects on human health [40,41]. The significance of literacy in relation to planetary health has been previously delineated, emphasizing the importance of an individual and societal approach based on educational programs [41], but the connection to health psychology is still an area to be explored [7].

Health psychologists possess the expertise to address the behavioral challenges associated with climate change [12,13,14]. Their proficiency in behavior change mechanisms, intervention design, and public health initiatives can be leveraged within interdisciplinary research to develop effective climate mitigation strategies [12]. A key aspect of health psychology’s contribution is the identification of behaviors that are instrumental in mitigating the effects of climate change. A key contribution of health psychology is the identification of behaviors instrumental in mitigating climate change, many of which align with established health-promoting behaviors [12,13]. Given that achieving global emission reduction targets necessitates significant behavioral modifications, adapting to climate change requires profound shifts in both individual lifestyles and societal structures [13].

An interdisciplinary approach is essential to understanding and addressing climate-related health challenges. This requires identifying behaviors that are both beneficial and detrimental to environmental sustainability [40]. Certain pro-environmental behaviors, such as purchasing energy-efficient appliances, involve one-time actions, whereas others, such as waste separation, require sustained behavioral adherence. Implementing behavioral triggers that encourage the adoption of pro-environmental behaviors while discouraging harmful ones is critical. The continued exploitation of environmental resources, driven by economic incentives, convenience, and comfort, exacerbates pollution and climate change. Thus, effective behavioral change strategies must target the contingencies that sustain environmentally harmful behaviors, shifting them toward those that encourage pro-environmental actions. This effort requires interdisciplinary collaboration and supportive public policy [40].

Despite growing interest in behavioral interventions for climate action, psychological research in this area remains limited, with most studies focusing on short-term interventions rather than systemic, long-term solutions [15]. While individual actions are necessary, pro-environmental behavior change requires collective and systemic approaches that account for psychological, social, and political factors influencing human decision-making.

The relationship between health behaviors and climate outcomes is bidirectional [14]. This relationship manifests in several key dimensions. First, mitigating climate change’s adverse effects on human health necessitates adapting health behaviors to minimize negative environmental impacts. For instance, health interventions that inadvertently increase carbon emissions are counterproductive. Second, climate change exacerbates existing behavioral disparities across different social strata—within and between nations, across generations, and between genders. Addressing these disparities requires targeted interventions that consider contextual challenges at multiple levels. Health behaviors provide a crucial framework for understanding the false dichotomy between individual behavior change and systemic transformation. Pro-environmental behavior change is contingent upon the interplay of large-scale individual actions and coordinated political efforts. Climate change demands that health psychologists move beyond observational studies toward proactive engagement in reducing carbon footprints while simultaneously enhancing evidence-based healthcare services.

Recent research has compiled robust evidence demonstrating that psychological interventions can effectively promote public engagement in climate action [10]. However, scaling these interventions to drive meaningful societal change requires the integration of psychological, social, and political approaches. Effective behavioral change models must therefore be interdisciplinary, drawing on insights from behavioral science, economics, and policy research [10].

The role of emotions in behavior modification is often underestimated, particularly in the context of climate change. Emotions serve as both antecedents and consequences of behavior, creating a feedback loop in which sustainable actions generate positive emotions, reinforcing further engagement in pro-environmental behaviors [37]. Recognizing and incorporating emotional factors into behavioral interventions can enhance their effectiveness.

Addressing systemic barriers is essential to fostering an environment conducive to pro-environmental behavior. Many environmentally harmful behaviors are reinforced by existing infrastructures, market mechanisms, and socio-political structures. Consequently, interventions should not only target individual decision-making but also promote structural changes that make pro-environmental behavior more accessible, convenient, and socially reinforced [15].

One promising avenue for intervention is the use of behavioral nudges—subtle modifications in the environment that guide individuals toward sustainable choices without restricting autonomy. Examples include default enrollment in green energy programs, reminders to reduce waste, and financial incentives for climate-friendly consumer choices. However, while nudges can be effective, they should be complemented by policies that foster long-term habit formation and systemic changes, ensuring that pro-environmental behavior becomes the societal norm rather than the exception [40].

For pro-environmental behavior change to be effective, psychological research must move beyond individual-focused interventions and embrace a broader framework that integrates systemic solutions. Future research should explore how health psychology, behavioral economics, and policy design can be synergized to drive large-scale, lasting change [14]. The role of psychology in addressing climate change extends beyond understanding human behavior; it also involves designing interventions that promote collective adaptation and resilience.

The integration of interdisciplinary collaboration, psychological expertise, and policy-driven solutions provides a pathway to meaningful climate action that aligns individual health with planetary health [3]. The One Health approach offers a comprehensive framework for addressing climate change by shifting from crisis response to prevention through a holistic understanding of the problem.

## 4. Conclusions and Future Implications

The increasing impact of climate change on both physical and mental health underscores the critical role of health psychology in mitigating and adapting to these challenges. This article has examined how psychological science contributes to climate action by elucidating behavioral determinants, promoting sustainable practices, and developing interventions that enhance resilience and well-being. Health psychology provides key insights into stress responses, coping mechanisms, risk perception, and behavior change—factors that are essential in fostering adaptive responses to climate change [7,14,15].

Despite its potential, the integration of health psychology into climate action remains underutilized. Several barriers hinder its widespread application, including limited training among health professionals, insufficient interdisciplinary collaboration, and sociopolitical constraints that impede large-scale behavioral change [14,17]. Overcoming these obstacles requires a coordinated effort among psychologists, policymakers, public health officials, and environmental scientists. Strengthening the connection between psychology and climate science can enhance the efficacy of interventions, ensuring they are not only evidence-based but also scalable and actionable [7,27].

While behavioral science offers essential tools for climate mitigation and adaptation, several practical barriers remain:

- Scalability and accessibility of psychological interventions: How can climate-related psychological interventions be scaled up and made widely accessible, particularly in vulnerable populations? While small-scale studies have demonstrated the efficacy of behavior change interventions, their large-scale implementation remains a challenge due to limited funding, lack of infrastructure, and competing public health priorities [3,12].
- Overcoming climate denial and resistance to behavior change: A significant obstacle to climate action is the persistence of climate skepticism, misinformation, and resistance to behavioral change. Psychological research shows that values, identity, and political beliefs play a substantial role in shaping climate attitudes. Understanding how to design communication strategies that reduce resistance and promote engagement is crucial for fostering pro-environmental behavior [7,13].
- Integrating psychological strategies into public policies: Despite increasing recognition of the importance of behavioral change, policies often prioritize technological and economic solutions, overlooking the human factors that drive climate action. Ensuring that psychological insights are embedded into policy frameworks—such as urban planning, education, and public health initiatives—requires stronger advocacy and collaboration between psychologists and policymakers [5,10,14,27].

Addressing these barriers is essential for maximizing the impact of health psychology on climate action. Without systematic efforts to integrate psychological science into large-scale climate strategies, many behavior change interventions will remain fragmented and ineffective [42].

As the climate crisis accelerates, health psychology must adapt and expand its role in addressing its multifaceted impacts. Future research should prioritize the following:

1. Understanding the psychological determinants of pro-environmental behavior: Identifying the most effective motivators for climate action, particularly in different cultural, economic, and demographic contexts [13,17].
2. Developing trauma-informed approaches for climate anxiety and distress: The rise in climate anxiety and ecological grief necessitates targeted psychological interventions that help individuals and communities build resilience while maintaining engagement in climate action [25,26].
3. Integrating behavioral science into climate governance: Collaboration between psychologists, environmental scientists, and policymakers must be strengthened to ensure that behavioral insights inform large-scale climate policies [11,27].
4. Promoting systemic and collective action: Shifting the focus from individual behavior change to broader structural and societal transformations, recognizing that lasting climate action requires systemic interventions in addition to personal choices [15,40].
5. Advancing interdisciplinary research on climate and mental health: Exploring the long-term effects of climate change on psychological well-being and identifying strategies to mitigate its impact on at-risk populations [14,24].

In conclusion, health psychology plays a crucial yet underutilized role in addressing climate change. The psychological dimensions of climate change—spanning individual behavioral modification to broader societal adaptation—must be more comprehensively integrated into climate science and policy frameworks [7,15].

By fostering interdisciplinary collaborations, advocating for climate-sensitive mental health strategies, and designing interventions that align human behavior with environmental sustainability, psychology can contribute to a more resilient and adaptive society [3,12].

The urgency of the climate crisis demands not only scientific advancements but also actionable solutions that empower individuals and communities to engage in meaningful and sustainable climate action. Future research and policy efforts must prioritize the intersection of behavioral science, public policy, and environmental sustainability to develop comprehensive and effective strategies for mitigating climate change.

## Figures and Tables

**Table 1:** Key psychological constructs related to the climate crisis.

Concept ^1^	Description
Acute stress reactions and PTSD	Trauma-related responses to extreme, life-threatening weather events
Chronic stress	Ongoing psychological pressure caused by long-term environmental degradation
Climate anxiety	Fear and helplessness related to awareness of climate change
Existential crisis	Psychological disruption linked to future uncertainty and planetary decline
Ecological grief	Sorrow and loss due to environmental degradation and biodiversity loss
Acute stress and PTSD	Trauma-related responses to extreme, life-threatening weather events

^1^ Terms compiled from definitions and conceptual discussions in the cited literature (refs. [5,6,10,27,33]).

**Table 2:** Climate-related events and associated psychological outcomes.

Type of Climate Event	Examples	Associated Psychological Effects ^1^
Acute events	Hurricanes, wildfires, and floods	PTSD, acute stress, anxiety, and sleep disturbances
Sub-acute events	Heatwaves and droughts	Aggression, mood disturbances, cognitive impairments, and elevated suicide risk
Persistent climate change	Sea-level rise, prolonged warming, and land loss	Chronic stress, anxiety, ecological grief, and existential distress

^1^ Based on empirical studies ([5,6,10,14,17,26,27,29,30]).

**Table 3:** Key psychological and social factors influencing climate risk perception and adaptive behavior.

Key-Factor ^1^	Description
Climate adaptation and risk perception	Risk perception and perceived adaptive capacity influence adaptive behaviors
Variables and adaptation	Trust in government, climate knowledge, place attachment, and perceived responsibility impact adaptation
Determinants of adaptive behavior	Negative affect, descriptive norms, self-efficacy, and response efficacy are predictors of adaptation
Experience and climate knowledge	Prior experience with climate events and climate knowledge weakly correlate with adaptation
Cognitive biases and climate change	People prioritize short-term risks over long-term ones and focus on extreme weather events
Emotional and ideological barriers	Climate change challenges personal security, ideology, and religious beliefs
Pluralistic ignorance	Social groups fail to recognize and address climate change collectively
Values and emotion in behavior	Negative emotions arise when values are threatened, affecting engagement in climate action
Climate mitigation and adaptation	Perceived human-driven climate change increases support for mitigation and adaptation

^1^ The information was selected from peer-reviewed sources cited in the text [10,18,29,40], summarizing key findings in climate psychology, risk perception, and adaptive behavior.

## Data Availability

No new data were created or analyzed in this study. Data sharing is not applicable to this article.

## References

[1] Frazier L.D. (2020). The past, present, and future of the biopsychosocial model: A review of The Biopsychosocial Model of Health and Disease: New philosophical and scientific developments by Derek Bolton and Grant Gillett. New Ideas Psychol..

[2] Engel G.L. (1977). The need for a new medical model: A challenge for biomedicine. Science.

[3] Zhang R., Tang X., Liu J., Visbeck M., Guo H., Murray V., Mcgillycuddy C., Ke B., Kalonji G., Zhai P. (2022). From concept to action: A united, holistic and One Health approach to respond to the climate change crisis. Infect. Dis. Poverty.

[4] Nielsen K.S., Marteau T.M., Bauer J.M., Bradbury R.B., Broad S., Burgess G., Burgman M., Byerly H., Clayton S., Espelosin D. (2021). Biodiversity conservation as a promising frontier for behavioural science. Nat. Hum. Behav. Nat. Res..

[5] Thoma M.V., Rohleder N., Rohner S.L. (2021). Clinical Ecopsychology: The Mental Health Impacts and Underlying Pathways of the Climate and Environmental Crisis. Front. Psychiatry.

[6] Palinkas L.A., Wong M. (2020). Global climate change and mental health. Curr. Opin. Psychol..

[7] Santos O., Virgolino A., Vaz Carneiro A., de Matos M.G. (2021). Health Behavior and Planetary Health. Eur. Psychol..

[8] Adams M. (2021). Critical psychologies and climate change. Curr. Opin. Psychol..

[9] Matarazzo J.D. (1980). Behavioral health and behavioral medicine: Frontiers for a new health psychology. Am. Psychol..

[10] Clayton S. (2019). Psychology and climate change. Curr. Biol..

[11] Bernard P., Chevance B. (2023). Health psychology and climate change: A race against time Position Paper. Eur. Health Psychol..

[12] Talukder B., Ganguli N., Matthew R., vanLoon G.W., Hipel K.W., Orbinski J. (2021). Climate change-triggered land degradation and planetary health: A review. Land Degrad. Dev..

[13] Doell K.C., Todorova B., Vlasceanu M., Bak Coleman J.B., Pronizius E., Schumann P., Azevedo F., Patel Y., Berkebile-Wineberg M.M., Brick C. (2024). The International Climate Psychology Collaboration: Climate change-related data collected from 63 countries. Sci. Data.

[14] Clayton S. (2020). Climate anxiety: Psychological responses to climate change. J. Anxiety Disord..

[15] Papies E.K., Nielsen K.S., Soares V.A. (2024). Health psychology and climate change: Time to address humanity’s most existential crisis. Health Psychol. Rev..

[16] Whitmarsh L., Poortinga W., Capstick S. (2021). Behaviour change to address climate change. Curr. Opin. Psychol..

[17] Chevance G., Fresán U., Hekler E., Edmondson D., Lloyd S.J., Ballester J., Litt J., Cvijanovic I., Araújo-Soares V., Bernard P. (2023). Thinking Health-related Behaviors in a Climate Change Context: A Narrative Review. Ann. Behav. Med..

[18] Freschi G., Menegatto M., Zamperini A. (2023). How Can Psychology Contribute to Climate Change Governance? A Systematic Review. Sustainability.

[19] Araos M., Jagannathan K., Shukla R., Ajibade I., Coughlan de Perez E., Davis K., Ford J.D., Galappaththi E.K., Grady C., Hudson A.J. (2021). Equity in human adaptation-related responses: A systematic global review. One Earth.

[20] Freschi G., Menegatto M., Zamperini A. (2024). Conceptualising the Link between Citizen Science and Climate Governance: A Systematic Review. Climate.

[21] Smith T.W., Orleans C.T., Jenkins C.D. (2004). Prevention and Health Promotion: Decades of Progress, New Challenges, and an Emerging Agenda. Health Psychol..

[22] Johnson B.T., Acabchuk R.L. (2018). What are the keys to a longer, happier life? Answers from five decades of health psychology research. Soc. Sci. Med..

[23] Ogden J. (2012). Health Psychology: A Textbook.

[24] Lazarus R.S., Folkman S. (1987). Transactional theory and research on emotions and coping. Eur. J. Pers..

[25] Koolhaas J.M., Bartolomucci A., Buwalda B., de Boer S.F., Flügge G., Korte S., Meerlo P., Murison R., Olivier B., Palanza P. (2011). Stress revisited: A critical evaluation of the stress concept. Neurosci. Biobehav. Rev..

[26] Ebi K.L., Hess J.J. (2020). Health Risks Due To Climate Change: Inequity In Causes And Consequences. Health Aff..

[27] Schwartz S.E.O., Benoit L., Clayton S., Parnes M.K.F., Swenson L., Lowe S.R. (2023). Climate change anxiety and mental health: Environmental activism as buffer. Curr. Psychol..

[28] Cunsolo A., Ellis N.R. (2018). Ecological grief as a mental health response to climate change-related loss. Nat. Clim. Change.

[29] Steg L. (2024). Psychology of Climate Change. Annu. Rev. Psychol..

[30] Stevens L.M., Burke A.E., Golub R.M. (2012). Posttraumatic Stress Disorder. JAMA.

[31] Blevins C.A., Weathers F.W., Davis M.T., Witte T.K., Domino J.L. (2015). The posttraumatic stress disorder checklist for DSM-5 [PCL-5]: Development and initial psychometric evaluation. J. Trauma Stress.

[32] Weathers F.W., Litz B.T., Keane T.M., Palmieri P.A., Marx B.P., Schnurr P.P. (2013). The PTSD Checklist for DSM-5 [PCL-5].

[33] Ma T., Moore J., Cleary A. (2022). Climate change impacts on the mental health and wellbeing of young people: A scoping review of risk and protective factors. Soc. Sci. Med..

[34] World Meteorological Organization (2024). El Niño-Linked Rains Trigger Devastation in Brazil. https://wmo.int/media/news/el-nino-linked-rains-trigger-devastation-brazil.

[35] World Meteorological Organization (2024). Devastating Rainfall Hits Spain in yet Another Flood-Related Disaster. https://wmo.int/media/news/devastating-rainfall-hits-spain-yet-another-flood-related-disaster.

[36] Whitmee S., Haines A., Beyrer C., Boltz F., Capon A.G., de Souza Dias B.F., Ezeh A., Frumkin H., Gong P., Head P. (2015). Safeguarding human health in the Anthropocene epoch: Report of The Rockefeller Foundation–Lancet Commission on planetary health. Lancet.

[37] Siegrist M., Árvai J. (2020). Risk Perception: Reflections on 40 Years of Research. Risk Analysis.

[38] Slovic P. (1987). Perception of Risk. Science.

[39] Cebulla A. (2007). Class or individual? A test of the nature of risk perceptions and the individualisation thesis of risk society theory. J. Risk Res..

[40] Schneider C.R., van der Linden S. (2023). An Emotional Road to Sustainability: How Affective Science Can Support pro-Climate Action. Emot. Rev..

[41] de Castañeda R.R., Villers J., Guzmán C.A.F., Eslanloo T., de Paula N., Machalaba C., Zinsstag J., Utzinger J., Flahault A., Bolon I. (2023). One Health and planetary health research: Leveraging differences to grow together. Lancet Planet. Health.

[42] Jochem C., von Sommoggy J., Hornidge A.K., Schwienhorst-Stich E.M., Apfelbacher C. (2023). Planetary health literacy: A conceptual model. Front. Public Health.

